# Positive and Negative Selection in the Thymus and the Thymic Paradox

**DOI:** 10.1155/1998/89415

**Published:** 1998

**Authors:** Julie N. Reza, Mary A. Ritter

**Affiliations:** Department of Immunology Imperial College School of Medicine Hammersmith Hospital Du Cane Road London W12 0NN United Kingdom

**Keywords:** Thymus, thymocyte maturation, positive selection, negative selection


*Developmental Immunology*, 1998, Vol. 5, pp. 161–168
Reprints available directly from the publisher
Photocopying permitted by license only

© 1998 OPA (Overseas Publishers Association)
Amsterdam B.V. Published under license
under the Harwood Academic Publishers imprint,
part of the Gordon and Breach Publishing Group
Printed in Malaysia

### Positive and Negative Selection in the Thymus and the Thymic Paradox

JULIE N. REZA MARY A. RITTER*

*Department of Immunology, Imperial College School of Medicine, Hammersmith Hospital, Du Cane Road, London W12 0NN*

*(Received 15 April 1997)*

*Keywords:* Thymus, thymocyte maturation, positive selection, negative selection

### INTRODUCTION

The development of T cells occurs in the thymus, with thymocytes undergoing phenotypic changes and acquiring their repertoire for the antigen-specific T-cell receptor (TcR; Blackman et al., 1988) during their migration through this organ. The TcR are generated by random genetic rearrangements/recombinations of V, D, and J elements, which, together with junctional diversity and novel insertions or deletion of bases at their V-D or D-J junctions, give rise to up to $10^9$ V-D-J genes in mice (Marrack and Kappler, 1986; 1988).

In the periphery, the resulting TcR interact with antigen-derived peptides presented by self-MHC on target cells. Due to the random mechanism by which they arise, however, it is possible that many of these TcR do not recognize the (antigen-presenting) self-MHC molecules or that they are autoreactive. These would be useless or even deleterious. Thus, developing thymocytes must be "educated," such that they are selected according to the specificity and affinity of the TcR that they express. This education involves deletion of autoreactive cells (negative selection), and "major histocompatibility complex (MHC) restriction" (i.e., selection of thymocytes able to recognize self-MHC molecules, called positive selection), and relies on the concept that whereas receptor-antigen binding might cause T-cell activation in the periphery, a similar interaction in the thymus may cause thymocyte death. Much regarding these selective processes remains unclarified, despite a vast cohort of research (reviewed by Fink and Bevan, 1995; Jameson et al., 1995; Anderson et al., 1996; Guidos, 1996).

### POSITIVE SELECTION

The first indication of self-MHC restriction came from Zinkernagel and Doherty (1975), who discovered that virus-specific $CD8^+$ T cells were unable to kill allogeneic virus-infected targets. The issue was then addressed using radiation-induced bone-marrow

*Corresponding author. Present address: Mary A. Ritter, Department of Immunology, Imperial College School of Medicine, Hammersmith Hospital, Du Cane Road, London W12 0NN.

chimeric mice (Bevan, 1977; Fink and Bevan, 1978; Zinkernagel et al., 1978). For instance, after using H-$2^b \times$ H-$2^d$ (F1) bone marrow to reconstitute lethally irradiated parental (P; H-$2^b$ or H-$2^d$) mice (such that chimeric animals had haemopoietic cells of b $\times$ d haplotype, whereas the radiation-resistant thymic components were either b or d haplotype), they found that $CD8^+$ T cells from the chimeras were restricted to the relevant parental strain. A similar outcome was observed for class II restricted responses. These results clearly indicated that positive selection was mediated by radiation-resistant stromal components, such as epithelium.

Use of deoxyguanosine (dGuo) to remove hemopoietic cells from fetal thymic lobes prior to grafting into a thymectomized animal also demonstrated that nonhemopoietic cells were responsible for restriction; this was confirmed using transgenic mice expressing genes for $D^b$/male H-Y antigen-specific TcRs (Lo and Sprent, 1986; Teh et al., 1988). T cells expressing the transgenic TcR were only efficiently selected on $D^b$ stromal cells (a nonselecting environment resulted in large numbers of immature DP cells and relatively small numbers of the mature SP cells). As both class I and II MHC are generally needed for CD8 and CD4 production (respectively), cortical epithelial cells, which express both MHC types and are resistant to dGuo/radiation, seemed to be the likely mediators, and indeed expression of class II molecules on defined thymic stromal subpopulations (Benoist and Mathis, 1989; Cosgrove et al., 1992; Laufer et al., 1996) support the view that it is the cortical epithelium that is responsible for positive selection.

Reaggregation assays have since shown that whereas fibroblasts are needed for DN to DP maturation, the cortical epithelium is necessary and sufficient for progress from DP to SP thymocytes (Anderson et al., 1993). The mechanism of epithelial cell action remains unclear, although the fact that 1-ethyl-1-3-(3′dimethyl-aminopropyl)carbodiimide (ECDI)-fixed class $II^+$ epithelial cells were effective in reaggregation assays suggests involvement of cell-surface molecules/surface-bound molecules, providing survival messages from apoptotic "death by neglect" (von Boehmer et al., 1989; Anderson et al., 1994). The inability of cortical epithelial cells to provide a second signal due to lacking molecules such as B7 might also be significant (Lorenz and Allen, 1989; Jenkinson et al., 1994).

Although the "point" at which T cells express TcR (i.e., $CD4^+8^+TcR^{low}$) might be thought to be the step at which positive selection occurs, it now seems that positive selection is a multistage phenomenon. The first step involves maturation of the $TcR^{low}$ subset to a DP $TcR^{high}$ subpopulation following interaction with the environment. Thus, DP $TcR^{high}$ cells are not found in TcR transgenic animals of nonselecting haplotypes whereas their DP $TcR^{low}$ precursors are (Ohashi et al., 1990; Swat et al., 1992). The putative second step of selection determines whether the cell turns out to be a $CD4^+$ or $CD8^+$ SP mature T cell. Again various hypotheses account for this. Originally, CD4 or CD8 commitment was thought to take place in one of two ways (reviewed by von Boehmer, 1994; Jameson et al., 1995). The instructive model postulated that interaction of DP cells with class II MHC resulted in downregulation of CD8, to produce $CD4^+$ SP cells, whereas interaction with class I produced $CD8^+$ cells. The stochastic/selective model, on the other hand, suggested DP cells randomly downregulated CD4 or CD8, irrespective of TcR specificity. If a given cell can then interact via TcR and its CD4 or CD8 coreceptor, it survives. If not, it dies (Figure 1). The selective/stochastic model was supported by the discovery in class I or class II deficient mice of so-called "transitional cells," which express the "wrong" coreceptor (Chan et al., 1993; Crump et al., 1993; Davis et al., 1993; van Meerwijk and Germain, 1993; Chan et al., 1994; Corbella et al., 1994; Pircher et al., 1994; Lundberg et al., 1995). Thus, $CD4^+CD8^{low}$ class I restricted cells have been found in class II knockout mice expressing transgenic TcRs specific for the H-Y antigen/class I (Chan et al., 1993). Similarly, $CD8^{high}CD4^{low}$ thymocytes could be found in class I deficient mice. Class I/II double-deficient mice showed none of these transitional subsets, indicating that interaction with MHC was needed in the process (Chan et al., 1993). These findings led to a modified model of stochastic/selective commitment. The first stage involves TcR of

DPs *interacting with MHC* resulting in random downregulation of CD4 or CD8, followed by a second step in which cells interact with MHC again, such that those that express inappropriate TcR die by neglect (reviewed by Fink and Bevan, 1995). Nevertheless, it is interesting to note that *in vitro*, Merkenschlager has shown that during positive selection, each thymocyte only interacts once with the stromal cell (Merkenschlager, 1996).

However, the debate does not end there. Further studies have prompted other hypotheses, including that of lineage precommitment or asymmetric commitment (instruction-dependent CD8 commitment but CD4 commitment by default; Crompton et al., 1994; Suzuki et al., 1995). The latter model was suggested after studies using a "reexpression" assay to overcome one of the major problems of following the commitment process—that termination of CD4 or CD8 synthesis does not affect CD4 or CD8 molecules already on the cell surface. Thus, cell-surface coreceptors were cleaved with pronase and subsequent CD4 and CD8 surface "reexpression" followed in suspension cultures. These experiments showed the presence of CD8 commited cells among $CD4^+CD8^{low}$ transitional cells (which would be expected to give rise to $CD4^+8^-$ SPs only). These cells in turn give rise to $CD4^{low}CD8^+$ transitional cells. CD4 commited cells, on the other hand, were only found in the $CD4^-CD8^{low}$ transitional pool. Further studies using this reexpression system suggested that commitment to the CD4 lineage does not require MHC class II expression or MHC class II dependent signals, particularly as CD4 commited thymocytes were even found in MHC class I and class II deficient mice

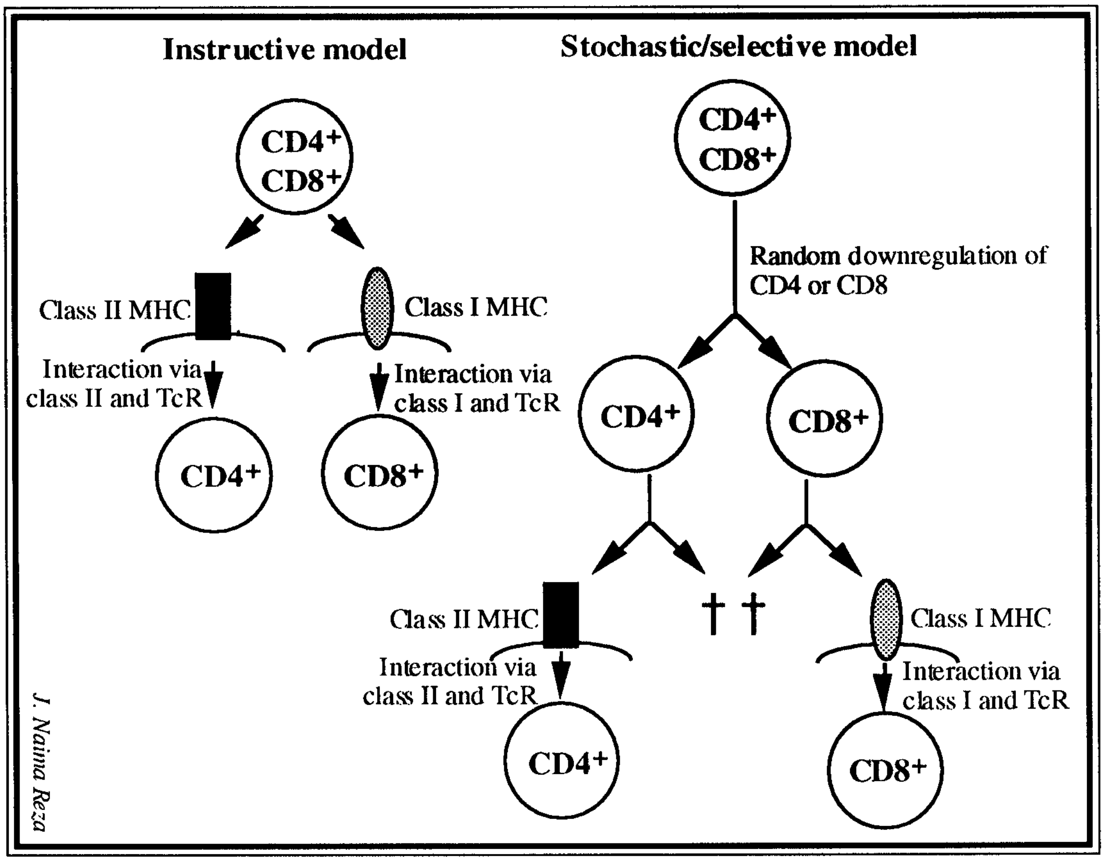

FIGURE 1 Schematic representation of the instructive model versus the stochastic/selective model.

(although interaction with atypical monomorphic MHC class II or low levels of class I was not discounted). The study also suggested that CD8 commitment signals preempt commitment to the CD4 lineage, as CD4 commited cells were deficient in animals expressing MHC class I restricted transgenic TcR in a positively selecting thymus, but not if in a nonselecting thymus. Overall, their results led them to conclude that CD8 lineage commitment requires class I expression and that CD8 commitment is postulated to be instructional, whereas CD4 commitment occurs by default. Such asymmetry has previously been suggested by Shortman's group (Lundberg et al., 1995), who found that a major proportion of the $CD4^{+}CD8^{int}TcR^{int}$-derived progeny of class II deficient mice, for instance, were $CD4^{-}CD8^{+}TcR^{high}$. This asymmetric model of development is also supported by temporal differences in production of $CD4^{+}$ or $CD8^{+}$ progeny from their precursors (Lucas et al., 1994; Lundberg et al., 1995). Yet another model has recently been postulated—the "complex coreceptor model," in which an initial TcR engagement results in TcR upregulation and downmodulation of *both* coreceptors; if the cells are not negatively selected, coreceptors can be reexpressed, producing $CD4^{+}CD8^{low}TcR^{int}$ cells (due to faster kinetics of CD4 reexpression), which then undergo lineage-specific differentiation resulting in selective suppression of CD4 or CD8 synthesis (Lucas and Germain, 1996). Despite these various models for lineage commitment, the molecular mechanisms by which the coreceptors affect development remain unclear, although elegant transgenic studies using a hybrid protein composed of a CD8 extracellular/transmembrane domain and a CD4 intracellular domain clearly suggest that the CD4 cytoplasmic tail, which is known to interact with the tyrosine kinase Lck, favors the development of CD4 lineage T cells (Itano et al., 1996).

### NEGATIVE SELECTION

The first indication of tolerization to self-Ags had come from Owen (1945), who found that dizygotic (nonidentical) cattle twins were tolerant of each others blood, suggesting ontogenic tolerance was taking place. Since then, many others have shown that thymocytes are selected so that they are not autoreactive (Sprent et al., 1975; Jenkinson et al., 1985). There are three possibilities for tolerance induction: T cells must be (1) clonally deleted, (2) anergized, or (3) suppressed. Using an Ab specific for V$\beta$17a (a component of the TcR$\beta$ chain specific for I-E + unknown super antigen), Kappler et al. (1987) found that the percentage of V$\beta$17a$^{+}$ SP thymocytes was greatly reduced in I-E expressing mice compared to I-E$^{-}$ controls. Levels of V$\beta$17a$^{+}$ cells at the DP thymocyte stage were similar, suggesting that deletion rather than anergy/suppression had taken place. This was verified in the transgenic H-Y system; female $D^{b}$ mice transgenic for the TcR specific for H-Y/$D^{b}$ had higher numbers of peripheral $CD8^{+}$ T cells than their male counterparts (which expressed the H-Y deleting Ag), again suggesting clonal deletion of $CD8^{+}$ thymocytes (Kisielow et al., 1988).

These and other data suggested that the deletion takes place between DP and SP stages, and by using transgenic techniques to express I-E in selected thymic compartments, it was shown that this negative selection occurs in the medulla or the C-M junction (Fowlkes et al., 1988), although in other models, it can occur in either the cortex or the medulla according to the experimental situation—perhaps reflecting the location/quantity of deleting antigen (Pircher et al., 1989; Wack et al., 1996). By using dGuo-treated chimeric thymuses, it was noted that tolerance was only induced to the same MHC as the donor stem cells (and not to that of the thymic epithelium). Together with other studies, this suggested that MHC class II$^{+}$ bone-marrow-derived cells, such as DCs and macrophages, mediate deletion (Sprent et al., 1975; Jenkinson et al., 1985; Kappler et al., 1987; Marrack et al., 1988). It is believed that these bone-marrow-derived cells present self-Ags to the developing thymocytes, and that thymocytes that recognize the Ag are then deleted by apoptosis.

Some evidence suggests that the positive selection/epithelium and negative selection/bone-marrow-derived cell link is not absolute, although the mechanism involved may differ with cell type (Bix and Raulet, 1992; Vandekerckhove et al., 1992; Elliott, 1993). For instance, *in vitro* cell types, including immortalized cortical epithelium (Tanaka et al., 1993) and fibroblasts, are able to induce Ag-dependent deletion of DP thymocytes, although the dose of Ag required is influenced by ICAM-1 (Pircher et al., 1993). In addition, reaggregate cultures using different stromal-cell populations have shown that dendritic cells, cortical epithelial cells, and medullary epithelial cells can all cause deletion, whereas macrophages cannot (Volkmann and Stockinger, 1997).

In the periphery, thymic negative selection is supported by T-cell anergy or ignorance, or mediated by functional "suppression" (as seen in $T_h1/T_h2$ cell cross-regulation) and "infectious tolerance"; a transplantation phenomenon where T cells from mice tolerized to grafts using anti-CD4 and CD8 mAbs are able to tolerize naive lymphocytes (Green and Webb, 1993; Qin et al., 1993; Abbas et al., 1996); these overcome problems of "leakiness" and of tolerization to nonthymic self-Ags.

### THE THYMIC PARADOX

Processes of positive and negative selection present a paradox, whereby interaction with MHC/Ag can result in both positive selection (survival) and negative selection (death). Three main hypotheses have been put forward to account for this paradox:

### The Altered Ligand Model

This idea, suggested by Marrack and Kappler (1987), postulates that MHC/peptide ligands found on thymic epithelium might differ from those found elsewhere. The hypothesis is based on the premise that mature T cells never reenter the thymus, where they then could be activated upon epithelium reencounter. Although there was some initial data suggesting a difference in antigen-processing capacity between thymic epithelium and other antigen-presenting cells, peptide elution studies (Marrack et al., 1993; Rammensee et al., 1993) did not support it. Moreover, recent studies in mice with a single MHC/peptide show that this simple ligand can lead to the positive selection of a wide repertoire of thymocytes (Ignatowicz et al., 1996).

### The Differential Avidity Model

This hypothesis proposes that thymocytes with low avidity for MHC/self-peptide are (positively) selected, whereas those interacting with high avidity are deleted. Thus, there is a "window" of avidity (which takes account of the combination of affinity and ligand density) that allows thymocyte survival (Lo et al., 1986; Lo and Sprent, 1986). This window may shift with development, and the avidity of interaction will also reflect T-cell-receptor density, density of coreceptors, and density of MHC/peptide on the presenting thymic stromal cell. In general, it appears that thymocytes bearing TcR specific for a certain class I/peptide combination can be positively selected by low concentrations or analogs of peptides that would otherwise result in their death; for example, introduction of a CD8 transgene, analogous to an increase in TcR affinity, increases deletion (Robey et al., 1992; Allen, 1994; Ashton-Rickardt and Tonegawa, 1994; Hogquist et al., 1994; Sebzda et al., 1994; Ignatowicz et al., 1996). In the context of "TcR affinity," it is also interesting to note that recent studies using biosensors to study kinetics of TcR/ligand interactions demonstrate that the "window" of avidity resulting in positive selection is much lower than that for negative selection (Alam et al., 1996).

### The Qualitative Signal Model

This is the latest of the three main models (Janeway et al., 1989; Mannie, 1991; Janeway, 1995), and accounts for how different TcR-MHC/peptide interactions could lead to different outcomes. It is based on the idea that interaction with a selecting ligand results in qualitatively different signals to those following interaction with a negatively selecting ligand. Evidence supporting a different intracellular signal transduction pathway for positive versus negative selection came from studies such as those by Anderson and colleagues showing cyclosporin A selectively inhibits positive selection in reaggregate cultures (Anderson et al., 1995), and at a molecular level, for instance, it appears that the mitogen-activated protein kinase (MAPK) is crucial to positive selection and is irrelevant for negative selection (Alberola-Ila et al., 1996). Furthermore, positive selection of thymocytes in H-Y TcR was severely reduced in the presence of dominant negative $p21^{ras}$, a molecule believed to relay tyrosine kinase-derived signals (Swan et al., 1995).

Thus, it can be seen that there is much still to be learned about the processes of T-cell development. The topics discussed here form the basis of much current work in the field of thymopoiesis; and the mechanisms, molecules, and microenvironmental conditions involved in selection remain the subject of considerable controversy.

### *References*

Abbas A. K., Murphy K. M. and Sher A. (1996) Functional diversity of helper T lymphocytes. *Nature*, **383**, 787-793.

Alam S. M., Travers P. J., Wung J. L., Nasholds W., Redpath S., Jameson S. C. and Gascoigne N. R. J. (1996) T cell receptor affinity and thymocyte selection. *Nature*, **381**, 616-620.

Alberola-Ila J., Hogquist K. A., Swan K. A., Bevan M. J. and Perlmutteer R. M. (1996) Positive and negative selection invoke distinct signaling pathways. *J. Exp. Med.*, **184**, 9-18.

Allen P. M. (1994) Peptides in positive and negative selection: A delicate balance. *Cell*, **76**, 593-663.

Anderson G., Anderson K. L., Conroy L. A., Hallam T. J., Moore N. C., Owen J. J. and Jenkinson E. J. (1995) Intracellular signaling events during positive and negative selection of CD4+ CD8+ thymocytes in vitro. *J. Immunol.*, **154**, 3636-3643.

Anderson G., Jenkinson E. J., Moore N. C. and Owen J. J. (1993) MHC class II-positive epithelium and mesenchyme cells are both required for T cell development in the thymus. *Nature*, **362**, 70-73.

Anderson G., Moore N. C., Owen J. J. T. and Jenkinson E. J. (1996) Cellular interactions in thymocyte development. *Annu. Rev. Immunol.*, **14**, 73-99.

Anderson G., Owen J. J., Moore N. C. and Jenkinson E. J. (1994) Characteristics of an in vitro system of thymocyte positive selection. *J. Immunol.*, **153**, 1915-1920.

Ashton-Rickardt P. G. and Tonegawa S. (1994) A differential avidity model for T cell selection. *Immunol. Today*, **15**, 362-366.

Benoist C. and Mathis D. (1989) Positive selection of the T cell repertoire: Where and when does it occur? *Cell*, **58**, 1027-1033.

Bevan M. J. (1977) In a radiation chimaera, host H-2 antigens determine immune responsiveness of donor cytotoxic cells. *Nature*, **269**, 417-418.

Bix M. and Raulet D. (1992) Inefficient positive selection of T cells directed by haematopoietic cells. *Nature*, **359**, 330-333.

Blackman M. A., Kappler J. W. and Marrack P. (1988) T cell specificity and repertoire. *Immunol. Rev.*, **101**, 5-19.

Chan S. H., Cosgrove D., Waltzinger C., Benoist C. and Mathis D. (1993) Another view of the selective model of thymocyte selection. *Cell*, **73**, 225-236.

Chan S. H., Waltzinger C., Baron A., Benoist C. and Mathis D. (1994) Role of coreceptors in positive selection and lineage commitment. *EMBO J.*, **13**, 4482-4489.

Corbella P., Moskophidis D., Spanopoulou E., Mamalaki C., Tolaini M., Itano A., Lans D., Baltimore D., Robey E. and Kioussis D. (1994) Functional commitment to helper T cell lineage precedes positive selection and is independent of T cell receptor MHC specificity. *Immunity*, **1**, 269-276.

Cosgrove D., Chan S. H., Waltzinger C., Benoist C. and Mathis D. (1992) The thymic compartment responsible for the positive selection of CD4+ T cells. *Int. Immunol.*, **4**, 707-710.

Crompton T., Hefti H., Lees R. K., Pircher H. and MacDonald H. R. (1994) CD4/CD8 lineage commitment in T cell receptor transgenic mice: Evidence for precommitment of CD4+CD8+ thymocytes. *Semin. Immunol.*, **6**, 249-256.

Crump A. L., Grusby M. J., Glimcher L. H. and Cantor H. (1993) Thymocyte development in major histocompatability complex-deficient mice: Evidence for stochastic commitment to CD4 and CD8 lineages. *Proc. Natl. Acad. Sci. USA*, **90**, 10739-10743.

Davis C. B., Killeen N., Crooks M. E. C., Raulet D. and Littman D. R. (1993) Evidence for a stochastic mechanism in the differentiation of mature subsets of T lymphocytes. *Cell*, **73**, 237-247.

Elliott J. I. (1993) Thymic selection reinterpreted. *Immunol. Rev.*, **135**, 226-242.

Fink P. J. and Bevan M. J. (1978) H-2 antigens of the thymus determine lymphocyte specificity. *J. Exp. Med.*, **148**, 766-775.

Fink P. J. and Bevan M. J. (1995) Positive selection of thymocytes. *Adv. Immunol.*, **59**, 99-133.

Fowlkes B. J., Schwartz R. H. and Pardoll D. M. (1988) Deletion of self-reactive thymocytes occurs at a CD4+ 8+ precursor stage. *Nature*, **334**, 620-623.

Green D. R. and Webb D. R. (1993) Saying the "S" word in public. *Immunol. Today*, **14**, 523-525.

Guidos C. J. (1996) Positive selection of CD4+ and CD8+ T cells. *Cur. Opin. Immunol.*, **8**, 225-232.

Hogquist K. A., Jameson S. C., Heath W. R., Howard J. L., Bevan M. J. and Carbone F. R. (1994) T cell receptor antagonist peptides induce positive selection. *Cell*, **76**, 17-27.

Ignatowicz L., Kappler J. and Marrack P. (1996) The repertoire of T cells shaped by a single MHC/peptide ligand. *Cell*, **84**, 521-529.

Itano A., Salmon P., Kioussis D., Tolaini M., Corbella P. and Robey E. (1996) The cytoplasmic domain of CD4 promotes the development of CD4 lineage cells. *J. Exp. Med.*, **183**, 731-741.

Jameson S. C., Hogquist K. A. and Bevan M. J. (1995) Positive selection of thymocytes. *Annu. Rev. Immunol.*, **13**, 93-126.

Janeway C. A. (1995) Ligands for the T cell receptor: Hard times for avidity models. *Immunol. Today*, **16**, 223-225.

Janeway Jr. C. A., Dianzani U., Portoles P., Rath S., Reich E. P., Rojo J., Yagi J. and Murphy D. B. (1989) Cross linking and conformational change in T cell receptors: Role in activation and in repertoire selection. *Cold Spring Harb. Symp. Quant. Biol.*, **54**, 657-666.

Jenkinson E. J., Anderson G., Moore N. C., Smith C. A. and Owen J. J. (1994) Positive selection by purified MHC class II+ thymic epithelial cells in vitro: Costimulatory signals mediated by B7 are not involved. *Dev. Immunol.*, **3**, 265-271.

Jenkinson E., Jhittay P., Kingston R. and Owen J. J. (1985) Studies of the role of the thymic environment in the induction of tolerance to MHC antigens. *Transplantation*, **39**, 331-333.

Kappler J. W., Roehm N. and Marrack P. (1987) T cell tolerance by clonal elimination in the thymus. *Cell*, **49**, 273-280.

Kisielow P., Bluthmann H., Staerz U. D., Steinmetz M. and von Boehmer H. (1988) Tolerance in T cell receptor transgenic mice involved deletion of nonmature CD4+8+ thymocytes. *Nature*, **333**, 742-748.

Laufer T. M., DeKoning J., Markowitz J. S., Lo D. and Glimcher L. (1996) Unopposed positive selection and autoreactivity in mice expressing class II MHC only on thymic cortex. *Nature*, **383**, 81-85.

Lo D., Ron Y. and Sprent J. (1986) Induction of MHC-restricted specificity and tolerance in the thymus. *Immunol. Res.*, **5**, 221-232.

Lo D. and Sprent J. (1986) Identity of cells that imprint H-2-restricted T cell specificity in the thymus. *Nature*, **319**, 672-675.

Lorenz R. G. and Allen P. M. (1989) Thymic cortical epithelial cells can present self antigens in vivo. *Nature*, **337**, 560-562.

Lucas B. and Germain R. N. (1996) Unexpectedly complex regulation of CD4/CD8 coreceptor expression supports a revised model for CD4+CD8+ thymocyte differentiation. *Immunity*, **5**, 461-477.

Lucas B., Vasseur F. and Penit C. (1994) Production, selection and maturation of thymocytes with high surface density of T cell receptor. *J. Immunol.*, **153**, 53-62.

Lundberg K., Heath W., Kontgen F., Carbone F. R. and Shortman K. (1995) Intermediate steps in positive selection: Differentiation of CD4+8int TCRint thymocytes into CD4-8+ TCRhi thymocytes. *J. Exp. Med.*, **181**, 1643-1651.

Mannie M. D. (1991) A unified model for T cell antigen recognition and thymic selection of the T cell repertoire. *J. Theor. Biol.*, **151**, 169-192.

Marrack P., Ignatowicz L., Kappler J. W., Boymel J. and Freed J. H. (1993) Comparison of peptides bound to spleen and thymus class II. *J. Exp. Med.*, **178**, 2173-2183.

Marrack P. and Kappler J. (1986) The antigen-specific, major histocompatability complex restricted receptor on T cells. *Adv. Immunol.*, **38**, 1-30.

Marrack P. and Kappler J. (1987) The T cell receptor. *Science*, **238**, 1073-1079.

Marrack P. and Kappler J. (1988) The T cell repertoire for antigen and MHC. *Immunol. Today*, **9**, 308-315.

Marrack P., Lo D., Brinster R., Palmiter R., Burkly L., Flavell R. H. and Kappler J. (1988) The effect of thymus environment on T cell development and tolerance. *Cell*, **53**, 627-634.

Merkenschlager M. (1996) Tracing interactions of thymocytes with individual stromal cell partners. *Eur. J. Immunol.*, **26**, 892-896.

Ohashi P. S., Pircher H., Burki K., Zinkernagel R. M. and Hengartner H. (1990) Distinct sequence of positive and negative selection implied by thymocyte T cell receptor densities. *Nature*, **346**, 861-863.

Owen R. D. (1945) Immunogenetic consequences of vascular anastomoses between bovine twins. *Science*, **102**, 400-401.

Pircher H., Brduscha K., Steinhoff U., Kasai M., Mizuochi T., Zinkernagel R. M., Hengartner H., Kyewski B. and Muller K. P. (1993) Tolerance induction by clonal deletion of CD4+8+ thymocytes in vitro does not require dedicated antigen-presenting cells. *Eur. J. Immunol.*, **23**, 669-674.

Pircher H., Burki K., Land R., Hengartner H. and Zinkernagael R. M. (1989) Tolerance induction in double specific T cell receptor transgenic mice varies with antigen. *Nature*, **342**, 559-561.

Pircher H., Ohashi P. S., Boyd R. L., Hengartner H. and Brduscha K. (1994) Evidence for a selective and multi-step model of T cell differentiation: CD4 + CD8 low thymocytes selected by a transgenic T cell receptor on major histocompatibility complex class I molecules. *Eur. J. Immunol.*, **24**, 1982-1987.

Qin S., Cobbold S. P., Pope H., Elliot J., Kioussis D., Davies J. and Waldmann H. (1993) "Infectious" transplantation tolerance. *Science*, **259**, 974-977.

Rammensee H. G., Falk K. and Rotzschke O. (1993) MHC molecules as peptide receptors. *Cur. Opin. Immunol.*, **5**, 35-44.

Robey E. A., Ramsdell F., Kioussis D., Sha W., Loh D., Axel R. and Fowlkes B. J. (1992) The level of CD8 expression can determine the outcome of thymic selection. *Cell*, **69**, 1089-1096.

Sebzda E., Wallace V. A., Mayer J., Yeung R. S., Mak T. W. and Ohashi P. S. (1994) Positive and negative thymocyte selection induced by different concentrations of a single peptide. *Science*, **263**, 1615-1618.

Sprent J., von Boehmer H. and Nabholz M. (1975) Association of immunity and tolerance to host H-2 determinants in irradiated F1 hybrid mice reconstituted with bone marrow cells from one parental strain. *J. Exp. Med.*, **142**, 321-331.

Suzuki H., Punt J. A., Granger L. G. and Singer A. (1995) Asymmetric signaling requirements for thymocyte commitment to the CD4+ versus CD8+ T cell lineages: A new perspective on thymic commitment and selection. *Immunity*, **2**, 413-425.

Swan K. A., Alberola-Ila J., Gross J. A., Appleby M. W., Forbush K. A., Thomas J. F. and Perlmutter R. M. (1995) Involvement of $p21^{ras}$ distinguishes positive and negative selection in thymocytes. *EMBO J.*, **14**, 276-285.

Swat W., Dessing M., Baron A., Kisielow P. and von Boehmer H. (1992) Phenotypic changes accompanying positive selection of CD4+CD8+ thymocytes. *Eur. J. Immunol.*, **22**, 2367-2372.

Tanaka Y., Mamalaki C., Stockinger B. and Kioussis K. (1993) In vitro negative selection of alpha beta T cell receptor transgenic thymocytes by conditionally immortalised thymic cortical epithelial cell lines and dendritic cells. *Eur. J. Immunol.*, **23**, 2614-2621.

Teh H. S., Kisielow P., Scott B., Kishi H., Uematsu Y., Bluthmann H. and von Boehmer H. (1988) Thymic major histocompatibility antigens and the alpha beta T cell receptor determine the CD4/CD8 phenotype of T cells. *Nature*, **335**, 229-233.

Vandekerckhove B. A., Namikawa R., Bachetta R. and Roncarolo M. G. (1992) Human haemopoietic cells and thymic epithelial cells induce tolerance via different mechanisms in the SCID-hu mouse thymus. *J. Exp. med.*, **175**, 1033-1043.

van Meerwijk J. P. and Germain R. N. (1993) Development of mature CD8+ thymocytes: Selection rather than instruction? *Science*, **261**, 911-915.

Volkmann A., Zal T. and Stockinger B. (1997) Antigen presenting cells in the thymus that can negatively select MHC class II restricted T cells recognizing a circulating self antigen. *J. Immunol.*, **158**, 693-706.

von Boehmer H. (1994) Positive selection of lymphocytes. *Cell*, **76**, 219-228.

von Boehmer H., Teh H. S. and Kisielow P. (1989) The thymus selects the useful, neglects the useless and destroys the harmful. *Immunol. Today*, **10**, 57-61.

Wack A., Ladyman H. M., Williams O., Roderick K., Ritter M. A. and Kioussis D. (1996) Direct visualisation of thymocyte apoptosis in neglect, acute and steady state negative selection. *Int. Immunol.*, **8**, 1537-1548.

Zinkernagel R. H., Callahan G. N., Althage A., Cooper S. M., Klein P. A. and Klein J. (1978) On the thymus in the differentiation of "H-2 self-recognition" by T cells: Evidence for dual recognition. *J. Exp. Med.*, **147**, 882-896.

Zinkernagel R. H. and Doherty P. C. (1975) H-2 compatibility requirement for T cell-mediated lysis of target cells infected with lymphocytic choriomeningitis virus. Different cytotoxic T cell specificities are associated with structures coded for in H-2K or H-2D. *J. Exp. Med.*, **141**, 1427-1436.

